# Health systems strengthening, dissemination, and implementation science in Africa: *quo vadis*?

**DOI:** 10.11604/pamj.2021.40.51.30399

**Published:** 2021-09-21

**Authors:** Berjo Dongmo Takoutsing, Chibuikem Ikwuegbuenyi, Alice Umutoni, Oloruntoba Ogunfolaji, Nourou Dine Adeniran Bankole, Racheal Mpokota, Gideon Adegboyega, Ulrick Sidney Kanmounye, Yao Christian Hugues Dokponou

**Affiliations:** 1 Research Department, Association of Future African Neurosurgeons, Yaounde, Cameroon,; 2 Faculty of Health Sciences, University of Buea, Buea, Cameroon,; 3 College of Medicine and Health Sciences, University of Rwanda, Kigali, Rwanda,; 4 College of Medicine, University of Ibadan, Ibadan, Nigeria,; 5 Neurosurgery Department, Faculty of Medicine and Pharmacy, Mohammed V University of Rabat, Rabat, Morocco,; 6 Levy Mwanawasa Medical University, Lusaka, Zambia,; 7 Queen Mary University of London, Barts and The London School of Medicine, London, United Kingdom,; 8 Operation Smile Ghana, Accra, Greater Accra, Ghana

**Keywords:** Public health, health policy, developing countries, population health, goals

## Abstract

Implementing health-system strengthening policies remains a challenge in Africa. Past successes, predictable but unanticipated flaws, underutilization of health services, traditional medicine, global inequity and poor practice by local stakeholders are some of the reasons many African countries have made little progress towards attaining global health goals. As a result, Africa has the highest disease burden despite multiple efforts from the global health community. These raise the question: what has to change so that health systems strengthening efforts in Africa are successful?

## Commentary

The World Health Organization (WHO) defines a health system as “all organizations, people and actions whose primary intent is to promote, restore or maintain health” [1]. It divides health systems into six components: workforce, service delivery, information management, medical technologies (infrastructure), health financing, and leadership and governance [1]. There are numerous challenges to implementing healthcare policies, especially in Africa [1]. However, when health-system strengthening is done the right way using appropriate healthcare policies, it can help to attain sustainable development, reduce disease burden and increase life expectancy.

Successful health system strengthening efforts are based on local needs and resources, and are adapted to the cultural context of the local community [1]. Most health system strengthening efforts in Africa have been led or funded by external organizations [2]. There are advantages and disadvantages to the involvement of non-African organizations in health systems strengthening for Africa. Advantages include funding, technical know-how, while disadvantages include earmarking and publication bias [2]. The agenda set by funders often limits how much the programs can be adapted to the local population´s needs. The pressure to always look successful skews the literature in favor of positive publications. Programs fail for multiple reasons, and we can learn more from the failed programs than from the successful ones [2].

While it is true that some failures of health systems strengthening initiatives can be attributed to colonial and neocolonial practices [3], it is also important to highlight the role of local stakeholders in the current and future state of African health systems. Conversations about the deficiencies of local health systems and the roles of local stakeholders happen every day in the corridors and streets of Africa. People ask, “can we truly increase our human resources for health when physicians are unpaid for years while government officials embezzle funds and go abroad for all their health care issues?” [4] or “how can we meet our goals when decision-makers were chosen based on nepotism, tribalism, or sexism?”. Unfortunately, these questions have not been addressed systematically in African health policy literature.

Systems-level thinking holds that uncoordinated investment in each of the six components ignores the relations between the components. Most conversations have focused on the lack of funding which is important but we cannot afford to neglect the role of leadership and governance in health system strengthening. Previously, it was believed that economic growth was a prerequisite for health improvement. However, a recent study identified the importance of healthcare governance when promoting change in unfavorable economic circumstances. Notably, the transitional government of Ethiopia that came to power in 1991 inherited one of the poorest and least developed countries in Africa [5]. Years later, the country has achieved continued economic growth attributed largely to an innovative approach to creating a sustainable health workforce backed by full government support [5].

Stakeholder identification and engagement are equally key to holistic health system strengthening [1]. Historically, we have done a good job of engaging government officials, policymakers, and healthcare workers at referral hospitals [6]. However, we often neglect district-level and rural healthcare workers, patient groups, and the general public [6]. Engagement of diverse interest groups enriches discussions, increases accountability, and improves priority setting and resource allocation [1,6]. Examples of successful stakeholder identification and engagement include the Doris Duke Charitable Foundation´s (DDCF) bid to promote accessible and inclusive healthcare policies in Africa during which they established the African health initiative [7]. The foundation convened focal groups in 5 sub-Saharan countries to open up discourse and analyze the effect of health system strengthening on the overall health target populations and the health system itself [7]. The Population Health and Implementation Training (PHIT) partnerships were planned using a decentralized model where each partner country implemented a project where the health challenges of the target population were addressed in their unique way [7]. Participating countries approached the project using their previous experiences to their advantage., Of particular note, Ghana and Tanzania approached the project from the angle of surveillance using past experiences in their community-based and essential health service delivery; Mozambique built its partnership on the foundation of primary health care scale-up; Rwanda established a working system using its community and health infrastructure; and Zambia banked on 25 years of HIV prevention and control to strengthen its health system [7].

It is important to promote the exchange of experiences and sharing of resources between African countries. Although it is unrealistic for every African country to have a center of excellence for each speciality, the regionalization of specialized care is an attainable goal, even more so in the era of the continental free trade agreement. We must promote regional and continental investments through the African Union and other economic groups. A great example of this concept is the Africa Centers for Disease Control and Prevention (Africa CDC) and its management of the COVID-19 pandemic [8]. These investments should help contain medical tourism within economic regions and the continent. Other cross-border health collaborations may involve medical education, research, advocacy and governance (especially the development of disease treatment guidelines by continental professional societies) [9,10]. The Africa Centres for Disease Control and Prevention (CDC) also champions the African Health Strategy (AHS) 2016-2030. AHS focuses on standardization of health professional training, qualification and licensing, harmonization of public health legislation, regulatory and standard-setting mechanisms [9]. Another example is the Regional East Africa Community Health (REACH) policy initiative which serves as a bridge linking health researchers and policymakers with goals to improve people's health and health equity through more effective use and application of knowledge to strengthen policy and practice in some East African communities [10]. For these cross-border collaborations to work, we must break down organizational and administrative barriers so that the health caregivers can share human and financial resources with other organizations, conduct joint planning in and out of their homeland towards other African countries.

For health system strengthening implementation and dissemination efforts in Africa to be successful, there is a need to stop previous unsuccessful practices and adopt practices that are targeted, self-funded, equitable, proven to work, demonstrating regionalization of specialized care together with multidisciplinary cross-border health collaborations and those coordinated by stakeholders with good leadership and governance located at all levels of the health system. When done this way using appropriate healthcare policies, it can help attain sustainable development reducing disease burden, resulting in a significant increase in life expectancy and hence moving a big step ahead towards attaining global health goals.
